# Distribution of health-related physical fitness in Slovak population

**DOI:** 10.1186/s40064-015-1479-4

**Published:** 2015-11-10

**Authors:** Viera Bebcakova, Bibiana Vadasova, Peter Kacur, Jan Junger, Iveta Borzikova, Martin Zvonar, Marta Gimunova

**Affiliations:** Faculty of Sports, University of Presov, Ul. 17. novembra 13, 08116 Prešov, Slovak Republic; Faculty of Sport Studies, Masaryk University, Kamenice 5, 62500 Brno, Czech Republic

**Keywords:** HFZ standards, Rural & urban, FITNESSGRAM, IPAQ

## Abstract

Purpose of the study was to examine relationship between distribution of healthy fitness zone standards of high school students and their type of housing or area of residence. Study sample consisted of 684 students (284 boys, 400 girls) from urban and rural areas of the region Presov in the eastern part of Slovakia. Physical fitness was assessed by four tests: back-saver sit and reach, shoulder stretch, curl-ups and 90° push-ups. Differences by place of residence and types of housing were examined by correspondence analysis of two-dimensional tables with computing Chi square value at significance level *p* < 0.05. Urban students performed higher level of flexibility, abdominal and upper strength and endurance than rural ones. Boys and girls living in a flat reached higher level of flexibility and abdominal strength/endurance however, they performed worse in upper strength and endurance than those living in a house. Slovak adolescents seem to have a healthier profile in abdominal muscular fitness and upper body flexibility than in lower body flexibility. The relationship between distribution of healthy fitness zone standards and residence area or housing type was revealed only in lower body flexibility, upper strength and endurance of urban and rural girls.

## Background

Physical fitness is defined as the ability of body to function effectively, to enjoy leisure, to be healthy, to resist disease, and to cope with emergency situations. Physical fitness is used in two close meanings: health-related which state the health and well-being and skill-related which is more task-oriented based on the ability to perform specific aspects of sports or occupations (Hian et al. 2013).

Health-related components of physical fitness include body composition, cardiovascular fitness, flexibility, muscular endurance, and strength (Ganley et al. 2011). Agility, balance, coordination, power, reaction time, and speed are components of skill-related fitness (Hian et al. 2013).

The current emphasis in physical fitness has shifted from performance-related to health-related indicators. Health-related physical fitness has been viewed as a narrower concept focusing on the aspects of fitness that are related to day-to-day functioning and health maintenance (Ujević et al. 2013).

Along with the modernization of the world, most of the technologies nowadays have made people less active because they want to do something with little input but bring out more output so people are making less of the physical work and this resulting in the decrement of fitness (Hian et al. 2013).

Appropriate physical activity is one of the main determinants of fitness (Gutin et al. 2005; Ruiz et al. 2006). Living in areas distinguished by population size can be associated with differences in eating habits, access to sport facilities and opportunities for physical activity. This environmental exposure might determine lifestyle behavior and it might be associated with fitness levels (De Vries et al. 2007; Roemmich et al. 2006; Parks et al. 2003).

However, it is not entirely clear whether such factors can affect aspects of body composition and, therefore, physical fitness (Tsimeas et al. 2005; Zvonař et al. 2010).

The distribution of health-related physical fitness across the population is not homogenous and has been found to differ, for example, according to gender, socio-economic status and ethnicity, as well as area of residence (Brug et al. 2012).

Some studies examining differences in physical activity, physical fitness, and overweight among rural and urban children show that children from rural areas and small cities were more active than urban children (Joens-Matre et al. 2008).

However, contradictory reports have also been published in relation to physical fitness parameters in children living in urban and rural settings. In some cases, no differences have been identified in a range of fitness and motor skill measures between children from urban and rural areas (Krombholz 1997; Tsimeas et al. 2005).

Several authors reported that better living conditions have been shown to offer a potential advantage for improved physical fitness in urban compared with rural children (Rutenfranz et al. 1982; Reyes et al. 2003; Bathrellou et al. 2007).

On the contrary, urban residence has been linked to sedentary lifestyle due to lack of adequate space for play, concerns for safety, automatic transportation and computerization of many activities (Bathrellou et al. 2007).

Chillón et al (2011) reported that the differences between places of residence are country- and region-specific, and data from different countries are required to better understand the relationship between place of residence and fitness in youth.

Researchers (Huang et al. 2010; Pratt et al. 1999; Sallis et al. 2000) have pointed out that much additional work is needed on geographical differences as compared with other social factors such as gender, race and ethnicity in studies of physical activity.

Moreover, most experts suggest that targeted interventions for specific subpopulations are needed to successfully increase physical activity (Baranowski et al. 2003). Thus, examining specific subpopulations within a social ecological approach is clearly needed (Joens-Matre et al. 2008).

The trend of increasing obesity observed in youth is often paralleled by a stabilization or even decrease of physical fitness and capacity. This has prompted various national and international organizations to promote programs counteracting the motor and health degradation of youths (Wilczewski et al. 1996).

Information about the regional distribution of health physical fitness status is necessary in order to tailor public health interventions, because a number of behavioral health risks are established in late childhood and early adolescence, including sedentary behavior and lack of strenuous exercise (Ujević et al. 2013).

Building on previous studies engaged in an issue of the distribution of healthy fitness zone standards we look for the answer to the question if there is the relationship between area of residence and achievement of HFZ in adolescents living in eastern part of Slovakia.

An answer to the question would enable us to monitor progressive or negative trend in the distribution of HFZ in adolescents. At the same time we would indirectly point out to the health quality of the population.

The purpose of the study was to analyze the relationship between the distribution of healthy fitness zone standards of high school students and type of housing (flat-house) or area of residence (urban-rural) in eastern part of Slovakia.

## Methods

Present study is based on data collected by testing adolescents of 14 randomly selected high schools in rural and urban areas in eastern part of Slovakia. At each high school two classes were randomly selected for participating in this study. Fitness testing was carried out from October to November 2014.

### Participants

Data on physical fitness were collected from 684 students attending randomly selected 14 high schools in Presov region (eastern part of Slovakia). Of these, 284 were boys (41.52 %) and 400 were girls (58.48 %). Mean age was 17.2 ± 1.2 years.

### Measures

High school students were tested for abdominal strength and endurance using curl-up test, upper body strength and endurance using 90° push up test, flexibility using shoulder stretch test and back-saver sit and reach test which are included in the FITNESSGRAM test battery (Plowman and Meredith 2013).

The FITNESSGRAM (Cooper Institute 2007) is in complex focused on testing health related physical fitness (body composition, aerobic fitness, muscle strength and endurance, flexibility) and motoric tests which are part of it are reliable enough for individual diagnostics (Suchomel 2004).

Demographic data on the location of residence (living in an urban or a rural area) and type of housing (house, flat) were obtained via IPAQ questionnaire (long version), which students completed online in the INDARES system.

The long version of the International Physical Activity Questionnaire (IPAQ) can be used internationally to obtain comparable estimates of PA. The questionnaire consists of six sections with specific questions: physical activity at school, physical activity during transport, physical activity in house/flat, physical activity in free time, time spent by sitting and demographic data. The questionnaire (IPAQ) has also been tested for reliability and validity and used in a number of international research projects (Craig et al. 2003). INDARES system consisted of several modules (questionnaires) related to physical activity (IPAQ, MPAM-R, WHO-5 etc.). Gained data were collected within extensive research conducted by the Center for Kinanthropology Research of Faculty of Physical Culture in Olomouc, Czech Republic.

The translation of IPAQ questionnaire to Slovak language was carried out by two sport linguists using the method of back translation of English version. The Slovak translation was compared to Czech version which is standardised. Based on the Czech and Slovak language proximity and socio-cultural environment the standardisation of Slovak version was not carried out.

Definitions of urban and rural areas are inconsistent. They are based on variables such as distance from trading centers and cut off population sizes of 100,000, 50,000, and 10,000 inhabitants (Tsimeas et al. 2005).

In Slovakia, state specifies according to law what is considered to be a village (usually less than 2000 inhabitants) and what is a town (more than 2000 inhabitants). Participant who chose in the IPAQ questionnaire place to live with more than 2000 inhabitants was classified as one who lives in urban area and less than 2000 inhabitants as one who lives in rural area.

According to Slovak socio-demographic reality a flat (usually without gardens) as a type of housing is considered to be block of flats` part in towns while houses (great majority of them with gardens) are usually part of towns` suburbs or villages.

Participant’s legal representative (in the case when subject was younger than 18 years) or participants (in the case when subject was older than 18 years) received a verbal description of the study procedures before testing and completed a written informed consent. The study protocol was approved by the ethical committee of Masaryk University, Brno, Czech Republic. Measurements were taken according to the ethical standards of the Declaration of Helsinki (Harriss and Atkinson 2011).

### Data analyses

For statistical processing was used correspondence analysis of two-dimensional tables with computing Chi square value. Two-dimensional tables include following parameters: urban–rural (pass–fail); flat–house (pass–fail). An alpha level of *p* < 0.05 was used for all statistical tests.

## Results

To interpret results and to answer the research question we used summary tables in which data about housing type (flat, house) and residence area (urban, rural) of participants are presented. The influence of social factor on the distribution of healthy fitness zone (HFZ) standards was categorized in a dichotomous way (pass-fail) in four tests physical fitness test (PFT) of FITNESSGRAM. Chi square test was used to determine the significance of relationships between the distribution of HFZ standards and residence area or housing type.

### Curl-up test

**Fig. 1:**
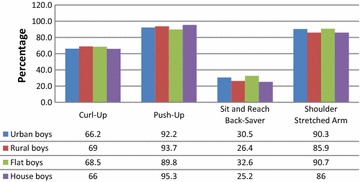
Distribution of healthy fitness zone standards in boys group according to the type of residence and housing

**Fig. 2:**
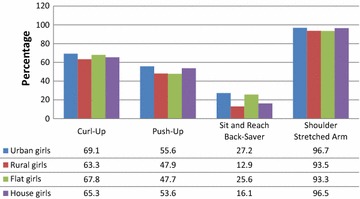
Distribution of healthy fitness zone standards in girls group according to the type of residence and housing

Results of the curl-up test are presented in Tables 1 and 2 for boys and girls, respectively. Of all participants, 100 urban boys (35.7 %) and 89 rural boys (31.8 %) met the standard (see Tables 1, 2). Results based on Chi square test showed that the distribution of HFZ standards did not correlate with an area of residence or type of housing. Regarding the fact that the number of boys and girls living in a town and in a village differed (same goes for living in a flat or a house), we evaluated collected data individually for urban (boys from town and girls from town) and rural areas (boys from village and girls from village) and analogically for living in a flat (boys from flat and girls from flat) and living in a house (boys living in house and girls living in house). These results are presented in Figs. 1 and 2 for all evaluated tests.

**Table 1:** Distribution of healthy fitness zone standards of boys in tests of FITNESSGRAM according to the type of residence and housing

Test	n	Urban				Rural				Chi square test
		Pass	%	Fail	%	Pass	%	Fail	%	
Curl-up	280	100	35.7	51	18.2	89	31.8	40	14.3	0.2428
Push-up	281	141	50.1	12	4.3	120	42.7	8	2.8	0.2675
BSR	283	47	16.6	107	37.8	34	12	95	33.6	0.5954
S-stretch	282	139	49.3	15	5.3	110	39	18	6.4	1.2638

Test	n	Flat				House				Chi square test
		Pass	%	Fail	%	Pass	%	Fail	%	
Curl-up	277	87	31.4	40	14.4	99	35.7	51	18.4	0.1954
Push-up	278	115	41.4	13	4.7	143	51.4	7	2.5	3.1172
BSR	280	42	15	87	31.1	38	13.6	113	40.3	1.8629
S-stretch	279	117	41.9	12	4.3	129	46.2	21	7.5	1.4675

*n* number of participants; % percentages; *BSR* back-saver sit and reach; *S-Stretch* shoulder stretched

**Table 2:** Distribution of healthy fitness zone standards of girls in tests of FITNESSGRAM according to the type of residence and housing

Test	n	Urban				Rural				Chi square test
		Pass	%	Fail	%	Pass	%	Fail	%	
Curl-Up	398	130	32.7	58	14.6	133	33.4	77	19.3	1.4967
Push-Up	400	105	26.2	84	21.0	101	25.3	110	27.5	2.3593*
BSR	322	41	12.7	110	34.2	22	6.8	149	46.3	10.4006**
S-Stretch	319	146	45.8	5	1.6	157	49.2	11	3.4	1.7483

Test	n	Flat				House				Chi square test
		Pass	%	Fail	%	Pass	%	Fail	%	
Curl-Up	397	101	25.4	48	12.1	162	40.8	86	21.7	0.2524
Push-Up	399	71	17.8	78	19.5	134	33.6	116	29.1	1.3226
BSR	326	31	9.5	90	27.6	33	10.1	172	52.8	4.3727*
S-Stretch	323	112	34.6	8	2.5	196	60.7	7	2.2	1.7640

*n* number of participants; % percentages; *significant relationship *p* < 0.05, **significant relationship *p* < 0.01; *BSR* back-saver sit and reach, *S-Stretch* shoulder stretched

The results showed that in 66.2 % of urban boys and 69 % of rural boys met HFZ standards for the curl-up test. Minimal differences in meeting HFZ standards were observed between boys living in a flat (68.5 %) and those living in a house (66 %).

Slightly bigger differences were found between urban and rural girls than in boys. In total, 130 urban girls (32.7 % of all participants) and 133 rural girls (33.4 % of all participants) met HFZ standards (see Tables 1, 2). Similarly to boys, results in the girls group showed that the distribution of HFZ standards did not correlate with an area of residence or type of housing.

Complete evaluation of HFZ standards distribution within urban and rural girls and girls living in a flat or a house is presented in Fig. 2. We found that 69.1 % of girls living in urban areas, 63.3 % of girls living in rural areas, 67.8 % of girls living in a flat and 65.3 % of girls living in a house met HFZ standards.

### Push-Up test

Results of the Push-Up test are presented in Tables 1 and 2 for both boys and girls, respectively. Of all participants, 141 boys living in urban areas (50.1 %) and 120 boys living in rural areas (42.7 %) reached HFZ standards. Similarly to previous results, statistical analysis revealed that the distribution of HFZ standards did not correlate with an area of residence or type of housing.

Complete evaluation of HFZ standards distribution for urban and rural boys and boys living in a flat or a house is presented in Fig. 1 together with other tests. Results of Push-Up test revealed that 92.2 % of urban boys and 93.7 % of rural boys met HFZ standards. According to the type of housing, 89.8 % of boys living in a flat and 95.3 % of boys living in a house reached HFZ standards. Boys showed high level of abdominal strength and endurance, which was confirmed by best results in Push-Up test of all four tests.

In contrast to boys, girls performed much worse than males. Only 105 urban girls (26.2 % of all participants) and 101 rural girls (25.3 % of all participants) met HFZ standards (see Tables 1, 2). Concerning the type of housing, 71 (17.8 %) girls living in a flat and 134 (33.6 %) girls living in a house reached HFZ standards.

Results of girls showed that the distribution of HFZ standards correlated with an area of residence (*p* < 0.05) in favor of urban girls. No correlation was found between the distribution of HFZ standards and type of housing.

Complete evaluation of HFZ standards distribution of urban and rural girls and girls living in a flat or a house is presented in Fig. 2. The results showed that 55.6 % of urban girls, 47.9 % of rural girls, 47.7 % of girls living in a flat and 53.6 % of girls living in a house met HFZ standards.

Whereas boys performed the best results in Push-Up test, girls achieved the second worst results in the test.

### Back-saver sit and reach test

Results of Push-Up test are presented in Tables 1 and 2 for boys and girls, respectively. Of all participants, 47 urban boys (16.6 %) and 34 rural boys (12 %) reached HFZ standards. In term of housing, 42 (15 %) boys living in a flat and 22 (6.8 %) boys living in a house met HFZ standards. Once again, the distribution of HFZ standards in boys group did not correlate with an area of residence or type of housing in back-saver sit and reach test.

Complete evaluation of HFZ standards distribution for urban and rural boys and boys living in a flat or a house is presented in Fig. 1 together with other tests. Results for back-saver sit and reach test showed that 30.5 % of urban, only 26.4 % of rural boys, 32.6 % of boys living in a flat and 25.2 % of boys living in a house met HFZ standards. Boys had very low level of flexibility and performed the worst in this flexibility test.

In back-saver sit and reach test, girls performed worse than boys. Only 41 urban girls (12.7 %) and 22 rural girls (6.8 %) of all participants reached HFZ standards (see Tables 1, 2). Regarding to the type of housing, 31 girls living in a flat (9.5 %) and 33 girls living in a house (10.1 %) met standards.

Results of girls showed that the distribution of HFZ standards correlated with an area of residence (*p* < 0.01) for urban girls group and with type of housing (*p* < 0.05) for girls living in a flat.

Complete evaluation of HFZ standards distribution for urban and rural girls and girls living in flat or house is presented in Fig. 2. The results showed that 27.2 % of urban girls, only 12.9 % of rural girls, 25.6 % of girls living in a flat and 16.1 % of girls living in a house met HFZ standards.

### Shoulder stretch test

Results of shoulder stretch test are presented in Tables 1 and 2. Of all participants, 110 urban boys (49.3 %) and 110 rural boys (39 %) reached HFZ standards. Regarding to the type of housing, 117 (41.9 %) boys living in a flat 129 (46.2 %) boys living in a house reached the HFZ standards. The distribution of HFZ standards in boys did not correlate with an area of residence or type of housing in back-saver sit and reach test.

Complete evaluation of HFZ standards distribution for urban and rural boys and boys living in flat or house is presented in Fig. 1 together with other tests. Results of shoulder stretch test revealed that 90.3 % of urban boys and 85.9 % of rural boys met HFZ standards. According to the type of housing, 90.7 % of boys living in a flat and 86 % of boys living in a house reached HFZ standards. Whereas the results of the first test measuring flexibility (back-saver sit and reach test) indicate very low level of flexibility, results in shoulder stretch test revealed contradictory findings.

Girls performed worse than boys in shoulder stretch test. Of all participants, 146 urban girls (45.8 %) and 157 rural girls (49.2 %) of all participants reached HFZ standards (see Tables 1, 2). Concerning the type of housing, 112 girls living in a flat (34.6 %) and 196 girls living in a house (60.7 %) met HFZ standards. No relationship was found between the distribution of HFZ standards and type of housing and area of residence.

Complete evaluation of HFZ standards distribution for urban and rural girls and girls living in flat or house is presented in Fig. 2. We found that 96.7 % of urban girls, 93.5 % of rural girls, 93.3 % of girls living in a flat and 96.5 % of girls living in a house reached HFZ standards. Within shoulder stretch test girls group performed the best results of all four tests.

## Discussion

Results point to minor differences in the level of abdominal strength and endurance between urban and rural students and students living in a flat or a house. Urban students performed better than those living in a rural area (1.5 %) as well as students living in a flat than students living in a house (2.5 %). Statistical analysis showed that there is no relationship between the distribution of HFZ standards and residence area or housing type in both males and females.

Minor differences in results are contrary to the results reported by Petroski et al. (2012) who found that adolescents from rural areas presented at almost 10 times higher chance of inadequate muscle strength/endurance than those from urban areas. Also, a study by Andrade et al. (2014) and Chillón et al. 2011) showed that with respect to residential location, urban adolescents had significantly higher mean score curl-ups (*p* < 0.01) than rural ones.

For upper body strength and endurance, urban students showed higher achievement rate percentage than rural students (3.1 % distinction). On the contrary, students living in a flat showed higher level upper body strength and endurance compared with students living in a flat (5.7 % distinction). Statistical analysis proved that the distribution of HFZ standards depends on type of housing only in females.

Urban adolescents showed higher level of flexibility than their rural counterparts (distinction in sit and reach back-saver test was 9.2 and 3.8 % in shoulder stretched arm test). Moreover, students living in a flat were more flexible than students living in a house (difference in sit and reach back-saver test was 8.45 % and only 0.75 % in shoulder stretched arm test). Apart from back-saver sit and reach test in which the relationship between the distribution of HFZ and type of housing and residence area was found, no other relationship was observed.

Study by Petroski et al. (2012) showed that with respect to flexibility, adolescents from urban areas presented a 56 % higher chance of inadequate flexibility than schoolchildren from rural areas. Similarly, Chillón et al. (2011) noted that rural young people had lower flexibility (*p* < 0.001) compared with their urban peers.

Generally, there are lots of studies around the world presenting contradictory results by comparing fitness levels of urban and rural children and adolescence. Tsimeas et al. (2005) have found that US urban children have superior fitness levels compare to those living in rural areas, whereas a report from Poland (Wilczewski et al. 1996) proposed that rural children were fitter than their urban counterparts. Chillón et al. (2011) presented results in which rural Spanish children and adolescents had overall a healthier profile than their urban peers in terms of upper- and lower-body muscular fitness, while they performed worse in speed-agility and flexibility.

One of the reasons for such discrepancy may be found in focusing on physical activity habits of urban and rural students in relation to the HFZ standards distribution. Huang et al. (2010) found no substantial differences in the physical activity habits and sedentary behaviors among students living in urban and rural areas where urban children reported more physical activity after school, on holidays and weekends, and also in total amount of physical activity compared with the rural children. Hence, public health awareness directed to enhance physical activity and decrease sedentary lifestyle among youngsters should focus equally to urban and rural children (Bathrellou et al. 2007).

Determining gender differences in the distribution of HFZ standards for abdominal strength and endurance has shown contradictory findings. Boys living in rural areas achieved higher level of percentage of HFZ standards distribution than girls (5.7 % difference). Girls living in a house or flat had lower success rate percentage of HFZ standards distribution than boys (0.7 % distinction for house or flat). Only girls living in urban areas were more successful in the HFZ standards distribution than boys (2.9 % distinction).

For the test which is a criterion of upper body strength and endurance most distinctive gender differences were found. Boys who live in urban and rural areas performed better than girls. Gender difference in urban areas was 36.6 and 45.8 % in rural areas. Similarly, girls living in a flat or house achieved worse scores than boys (42.1 % difference for flat and 41.7 % for house).

Similar results were reported by Andrade et al. (2014) in their study where boys showed significantly higher levels of strength and endurance and balance compared with girls.

Gender differences in flexibility present divergent results. In sit and reach back-saver test, boys who live in urban and rural area were better than girls (distinction 3.3 % in urban a 13.5 % in rural area). Similarly, boys living in a flat or a house performed better than girls (7 % distinction in flat and 9.1 % in a house). In shoulder stretch test, we observed better flexibility of upper body in favor of girls who live in urban and rural area (distinction 6.4 % in urban and 7.6 % in rural area) as well as in favor of girls living in a flat or a house (2.6 % difference for flat and 10.5 % for house). Andrade et al. (2014) reported findings contrary to our results as they found that boys showed significantly lower levels of the sit and reach test (*p* < 0.01).

Reyes et al. (2003) found that urban children of both sexes performed better in timed sit-ups and boys showed somewhat greater flexibility in the lower back and upper thighs (sit and reach) than girls. The differences in flexibility between urban and rural girls and urban and rural boys were not significant, which is similar to our results.

Renfrow et al. (2011) found that gender differences may be attributed to the difference of sport choice between boys and girls, especially in a private school setting.

What we should consider is the fact that only two thirds of students regardless of housing type or residence area (on average 66.9 %) reached an acceptable level of abdominal strength and endurance, which determines correct body posture.

Percentage success rate in the distribution of HFZ indicates that boys, regardless of housing type and residence area, showed on average 92.75 % achievement in the level of upper body strength and endurance. Girls did not meet standards and less than half of them (on average 51.2 %) achieved an acceptable level of upper body strength and endurance. Progressive muscle weaknesses in upper body may reflect in health quality and cause several muscle imbalances and disorders like upper crossed syndrome.

There were some discrepancies within the distribution of HFZ standards in flexibility tests. Adolescents performed significantly the worst results in back-saver sit and reach test (on average 28.68 % in boys and 20.45 % in girls) while in shoulder stretch test girls achieved the best results (on average 95 %) and boys the second best results (88.23 %).

Similarly, Dórea et al. (2008) found that only 51 % of the boys and 58 % of the girls in the sit-and-reach test reached the established criteria. Low level of flexibility in lower-limb area is negative predisposition for quality of life considering frequent occurrence of lower crossed syndrome.

There was on average 36 % of cases when girls and boys did not reach the HFZ standards in our study. Study of Powell et al. (2009) showed that 23 % did not meet the standard for muscular strength, endurance, and flexibility.

In conclusion, urban students performed better in majority of fitness tests than their counterparts living in rural areas. Similarly, Hian et al. (2013) observed that there were more urban students who had better score of physical fitness compared with the rural samples. It also could be related to Eiben et al. (2005) who noted that the urban boys and girls produced better physical performance than their rural counterparts. This may be caused by several factors reported by Loucaides et al. (2004) who found that equipment availability and transportations were better in urban than rural areas. Schools in urban areas also had better facilities such as field, track and others if compared with rural schools (Hian et al. 2013).

Physical fitness level in childhood and adolescence is positively associated with present and future health-related outcomes such as risk for obesity, cardiovascular disease, skeletal health and mental health (Ortega et al. 2008). Therefore, it is inevitable that a health-related physical education curriculum can provide students with substantially more physical activity during physical education classes. Sallis et al. (1997) point to the fact that improved physical education classes can potentially benefit 97 % of elementary school students.

The environment might have little influence on several health-related factors, since residence area and housing type differences were small for majority of tests. It is important to note that the place of residence and appropriate external motivation should be taken into account when implementing effective interventions to promote physical activity and health.

## Conclusions

Slovak adolescents seem to have a healthier profile in abdominal muscular fitness and upper body flexibility than in lower body flexibility. Boys performed better in all tests than girls apart from upper body flexibility.

Boys and girls from urban areas had higher level of flexibility, abdominal and upper strength and endurance than their rural counterparts. Those living in a flat reached higher level of flexibility and abdominal strength/endurance; however, they performed worse in upper strength and endurance than boys and girls living in a house.

The relationship between the distribution of HFZ standards and residence area or housing type was found only in lower body flexibility of girls and upper body strength and endurance of urban and rural girls.

## Abbreviations

HFZ: healthy fitness zone
IPAQ: International Physical Activity Questionnaire
PFT: physical fitness test
